# miR-22-3p/PGC1*β* Suppresses Breast Cancer Cell Tumorigenesis via PPAR*γ*

**DOI:** 10.1155/2021/6661828

**Published:** 2021-03-12

**Authors:** Xuehui Wang, Zhilu Yao, Lin Fang

**Affiliations:** ^1^Department of Thyroid and Breast Surgery, Shanghai Tenth People's Hospital, School of Medicine, Tongji University, Shanghai 200072, China; ^2^Nanjing Medical University, Nanjing 211166, China

## Abstract

In this study, we found that miR-22-3p expression was decreased in breast cancer (BC) cell lines and tissues. Overexpression of miR-22-3p inhibited the proliferation and migration of BC cells in vitro and in vivo, while depletion of miR-22-3p exhibited the opposite effect. Importantly, miR-22-3p could directly target PGC1*β* and finally regulate the PPAR*γ* pathway in BC. In conclusion, miR-22-3p/PGC1*β* suppresses BC cell tumorigenesis via PPAR*γ*, which may become a potential biomarker and therapeutic target.

## 1. Introduction

Breast cancer (BC) is the one of the most commonly diagnosed malignancies and the leading cause of cancer-related death in women [1]. Despite the fact that significant advances in surgical and medical management of BC have been exhibited, the incidence and mortality still increased by 18% since 2008 [2]. Higher rates of metastasis, recurrence, and drug resistance are the mainly reasons of poor prognosis and low survival among BC patients. Therefore, further investigating the molecular mechanism and discovery of the new biomarkers remains urgently needed for the diagnosis and treatment of BC.

MicroRNAs (miRNAs) are a class of single-stranded and highly conserved small noncoding RNAs, participating in numerous biological processes [3, 4]. miRNAs typically suppress gene expression at posttranscriptional levels by directly recognizing complementary sequences in the 3′untranslated region (3′-UTR) of target mRNAs. Various miRNAs have been identified to play significant roles in the etiology of BC. For example, miR-135-5p could inhibit TGF-*β*-induced epithelial-mesenchymal transition and metastasis by targeting SMAD3 in BC [5]. miR-27a facilitates BC progression via GSK-3*β* [6]. Specifically, low expression of serum miR-22 was found significantly associated with short survival and poor prognosis [7]. However, the role of miR-22 was demonstrated both as a tumor suppressor and a promoter in previous studies [8, 9].

As members of nuclear receptor superfamily, peroxisome proliferator-activated receptors (PPARs) are ligand-activated transcriptional factors (TFs). There are mainly three isotypes of PPARs, including PPAR*α*, PPAR*β*, and PPAR*γ* [10]. They are involved in cellular differentiation, cell proliferation, and tumorigenesis. Among them, increasing evidence suggests that PPAR*γ* protects against tumors by inhibiting cell proliferation. For example, PPAR*γ* could inhibit the development of lung adenocarcinoma through the regulation of tumor cell proliferation and transmission-related molecules [11, 12]. PPAR*γ* is prone to exert an antiangiogenic effect, which has been known as a hallmark of cancer [13]. Downregulation of PPAR*γ* is associated with decreased terminal differentiation and cell cycle arrest, which induces cell proliferation and leads to tumorigenesis [14].

Peroxisome proliferator-activated receptor gamma coactivators 1 alpha and beta (PPAGC1A/PGC1*α* and PPARGC1B/PGC1*β*, respectively) are major regulators of mitochondrial biogenesis and cellular metabolism [15, 16], playing important roles in the PPAR signaling network [17]. PGC1*β* has been proved to be associated with several cancers. For example, hepatic PGC1*β* acts as a transcriptional gatekeeper of mitochondrial function to contribute to hepatocellular carcinoma progression [18]. FOXO3/PGC1*β* signaling axis was proved essential to sustain the pancreatic ductal adenocarcinoma cancer stem cell properties [19]. Specifically, PGC1*β* was proved significantly overexpressed in BC and could inhibit the apoptosis of BC cells via the mTOR signaling pathway [20, 21]. PGC1*β* regulates HER2-overexpressing BC cell proliferation by metabolic and redox pathways [22]. PGC1*β* regulates BC tumor growth and metastasis by SREBP1-mediated HKDC1 expression [23]. In addition, PGC1*β* could cooperate with PPAR*γ*, allowing the subsequent interaction between PPAR*γ* and other transcription factors [24]. PGC1*β* mediates PPAR*γ* activation of osteoclastogenesis [25]. Therefore, we postulated that the PPAR signaling network plays an important role in the development and progression of BC.

In the present study, we found that miR-22-3p was downregulated in BC and suppressed BC cell tumorigenesis. Then, we demonstrated that PGC1*β* was regulated by miR-22-3p. Moreover, we found that the effects of miR-22-3p/PGC1*β* on BC were, at least in part, mediated by the PPAR*γ* signaling pathway.

## 2. Materials and Methods

### 2.1. Clinical Cancer Tissue Samples

Tumor tissues and their adjacent normal tissues of 47 BC patients were collected from the Department of Breast and Thyroid Surgery of Shanghai Tenth People's Hospital of Tongji University (Shanghai, China). None of the patients received any local or systemic treatment before surgery, and all tissue specimens were immediately snap-frozen in liquid nitrogen until further use. All studies in this manuscript were approved by Institutional Ethics Committees of Shanghai Tenth People's Hospital. We have obtained informed consent from all patients.

### 2.2. Cell Culture

The human HEK293T and human BC cell lines (MDA-MB-231, MCF-7, HCC-1937, and SKBR3) and normal breast epithelial cell line (MCF-10A) were obtained from Chinese Academy of Sciences (Shanghai, China). The HEK293T, MDA-MB-231, MCF-7, HCC-1937, and SKBR3 cells were cultured in Dulbecco's Modified Eagle's Medium (DMEM) (Gibco, USA) with 10% Fetal Bovine Serum (FBS) (Gibco, USA), penicillin (100 units/ml), and streptomycin (100 *μ*g/ml) (Enpromise, China). The MCF-10A cells were cultured in Mammary Epithelial Basal Medium (MEBM) (Cambrex, USA). All cells were cultured at 37°C with 5% CO_2_.

### 2.3. Transfection Assay

We purchased miR-22-3p mimics, miR-22-3p inhibitor, and nonspecific miR-negative control (miR-NC) oligo from RiboBio (Guangzhou, China). When the density of MDA-MB-231 or MCF-7 cells reached 80%, cells were transfected with 100 nmol/l miR-22-3p mimics, miR-22-3p inhibitor, or miR-NC using Hieff Trans™ Liposomal Transfection Reagent (Yeasen, China) according to the manufacturer's instructions. After 24-48 h of incubation, cells were harvested for further analysis.

### 2.4. Quantitative Real-Time Polymerase Chain Reaction (RT-qPCR)

Total RNA was extracted from frozen tissues and cultured cells by Trizol reagent (Invitrogen, Carlsbad, CA, USA), and the concentration and purity of RNA samples was assessed with a Nanodrop 2000 spectrophotometer (Thermo Fisher Scientific, USA). CDNA was synthesized by a commercial cDNA synthesis kit (Yeasen, China). We conducted RT-qPCR by using the SYBR Green PCR Kit (Yeasen, China), and primer sequences were designed and synthesized by RiboBio (Guangzhou, China). Expression of miRNAs was assessed by threshold cycle (CT) values and analyzed using the 2^-*ΔΔ*Ct^ method. The sequences of primers can be provided upon request.

### 2.5. MTT Assay

3-(4,5-Dimethylthiazol-2-yl)-2,5-diphenyltetrazolium bromide (MTT) assay was performed to detect cell proliferation ability. After 24 h transfection, a density of 2000 cells per well was placed into 96-well plates. The cells were detected in accordance with the manufacturer's instructions using 3-(4,5-Dimethylthiazol-2-yl)-2,5-diphenyltetrazolium bromide (MTT) assay kit (Sigma, Santa Clara, CA, USA). The 490 nm optical density was detected by a microplate reader (BioTek, USA).

### 2.6. Colony Formation Assay

A density of 800-1000 cells per well was transferred into 6-well plates. Cell colonies were washed twice by using cold phosphate-buffered saline (PBS), fixed with 75% ethanol, and stained with 0.1% crystalline purple until the colonies were visible. Then, colonies were photographed and counted.

### 2.7. Wound Healing Assay

MDA-MB-231 and MCF-7 cells were transfected with a range of constructs as indicated in 6-well plates. When the treated cells reached about 90% confluency, a scratch was produced in the cell monolayer by drawing a 200 *μ*l pipette tip over the surface of each well, holding the tip perpendicular to the plate. The monolayers were cultured in DMEM with 2% FBS. Pictures of wound healing were taken at 0 h and 24 h at the same position to observe cell movement.

### 2.8. Migration Assays

We used transwell chambers (Corning, Inc., Lowell, MA, USA) to measure the migration ability of the cells. Transfected cells were added into the upper chamber with 200 *μ*l serum-free medium, and medium with 10% FBS was added into the lower chamber. 12-24 h later, cells were removed in the upper chamber by cotton swab. Then, the cells on the opposite side of the filter were fixed with 75% ethanol for 10 min, then stained with 0.1% crystal violet for 10 min. Representative pictures were taken with a microscope (Leica Microsystems, Mannheim, Germany).

### 2.9. Dual-Luciferase Reporter Assay

According to our previous studies [26, 27], to confirm that miR-22-3p directly targets PGC1*β* 3′-UTR, wild and mutant reporter plasmids of PGC1*β* were individually designed and synthesized by IBSBio (Shanghai, China). HEK293T cells were cotransfected with the constructed reporter plasmids, together with miR-22-3p mimics or miR-22-3p-NC using Lipofectamine® 2000 (Invitrogen; Thermo Fisher Scientific, USA). 48 h later, the luciferase activities were measured with the Dual-Luciferase® Reporter Assay kit (Yeasen, China). Firefly to Renilla luciferase ratio was calculated.

### 2.10. Western Blotting Analysis

Proteins were extracted using RIPA lysis buffer (Beyotime, Jiangsu, China), and the concentrations were detected by using the protein assay kit (Beyotime, Jiangsu, China). Protein lysates were separated by 10% sodium dodecyl sulfate-polyacrylamide gels and then transferred to nitrocellulose membrane (Beyotime, Jiangsu, China), which was incubated 1 h with 5% nonfat milk and immunoblotted overnight at 4°C with primary antibodies: anti-PCNA (Proteintech, USA), anti-PGC1*β* (Abclonal, China), anti-PPAR*γ* (Abclonal, China), anti-NK-*κ*B (CST, USA), anti-C-myc (CST, USA), anti-MMP2 (CST, USA), anti-MMP9 (CST, USA), anti-cyclin D1 (Abcam, USA), and anti-cyclin E (Abcam, USA). The next day, the membranes were incubated in secondary antibodies for 1 h at room temperature. Dilutions of all antibodies used in this study were 1 : 1000. Signals of protein bands were scanned by Odyssey Infrared scanning system (Li-Cor, Lincoln, NE, USA).

### 2.11. FISH Assay

Ribo™ Fluorescent In Situ Hybridization Kit (Ribo, China) was used in FISH assay. Specific probes for the miR-22-3p were designed and synthesized by IBSBio (Shanghai, China). 4,6-Diamidino-2-Phenylindole (DAPI) was used to stain cell nuclei. A fluorescence microscope (Olympus BX53 Biological Microscope) was used to capture the images of cells.

### 2.12. Statistical Analysis

The significance of differences between groups was assessed by GraphPad Prism V8.3.0 (GraphPad, CA, USA). All experiments were repeated for three times. Data were obtained from three independent experiments which are presented as the means ± standard deviation (SD). Student's *t*-test (double-tailed) was used to draw a comparison between groups, and *p* value < 0.05 was considered significant.

## 3. Results

### 3.1. miR-22-3p Was Decreased in BC Cell Lines and Tissues

**Figure 1:**
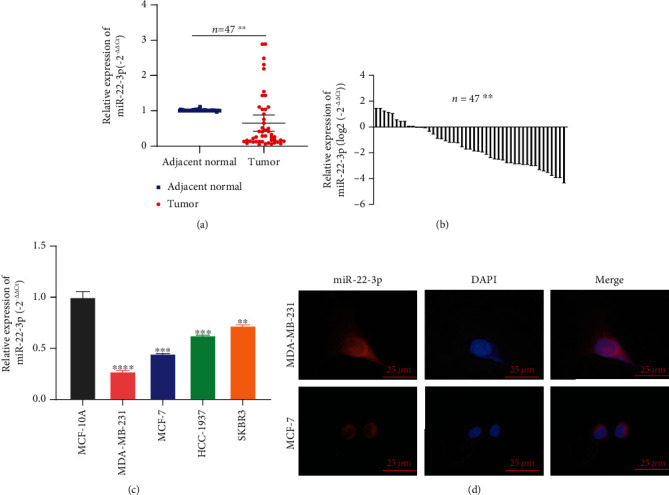
miR-22-3p was decreased in BC cell lines and tissues. (a, b) miR-22-3p had low expression in BC tissues compared with adjacent normal tissues. (c) miR-22-3p had low expression in BC cell lines. (d) Detection of colocalization of miR-22-3p in cytoplasm by RNA FISH assay (magnification, ×400). Red, miR-200a-3p; blue, DAPI. ^∗∗^*p* < 0.1; ^∗∗∗^*p* < 0.001; ^∗∗∗∗^*p* < 0.0001.

Results obtained from TGCA databases showed that expression of miR-22-3p was decreased in BC (Figure S1A). The expression of miR-22-3p was assessed by RT-qPCR in 47 pairs of BC tissues and adjacent normal tissues. Results of RT-qPCR showed that the expression of miR-22-3p was significantly decreased in BC tissues (35/47, 74.5%) (Figures 1(a) and 1(b)). In addition, we examined the expression of miR-22-3p in BC cell lines (MDA-MB-231, MCF-7, HCC-1937, and SKBR3) and normal breast epithelial cell line (MCF-10A). Consistent with the findings in BC specimens, the miR-22-3p expression was downregulated in BC cell lines (Figure 1(c)). To better explore the function and mechanism of miR-22-3p, RNA fluorescence in situ hybridization (FISH) analysis was performed to detect the localization of miR-22-3p. The FISH analysis revealed that miR-22-3p was mostly stained in the cytoplasm of BC cell lines (Figure 1(d)). After analyzing the relationship between the expression of miR-22-3p and the clinical pathological variables in 47 BC patients, we found that high expression of miR-22-3p was negatively associated with TNM stage, lymph node metastasis, and tumor size but had no correlation with age and distant metastasis (Table 1). The -2^*ΔΔ*ct^ value of miR-22-3p expression in BC tissues greater than that in adjacent normal tissues was considered high expression.

### 3.2. miR-22-3p Suppressed Cell Proliferation of BC Cells

**Figure 2:**
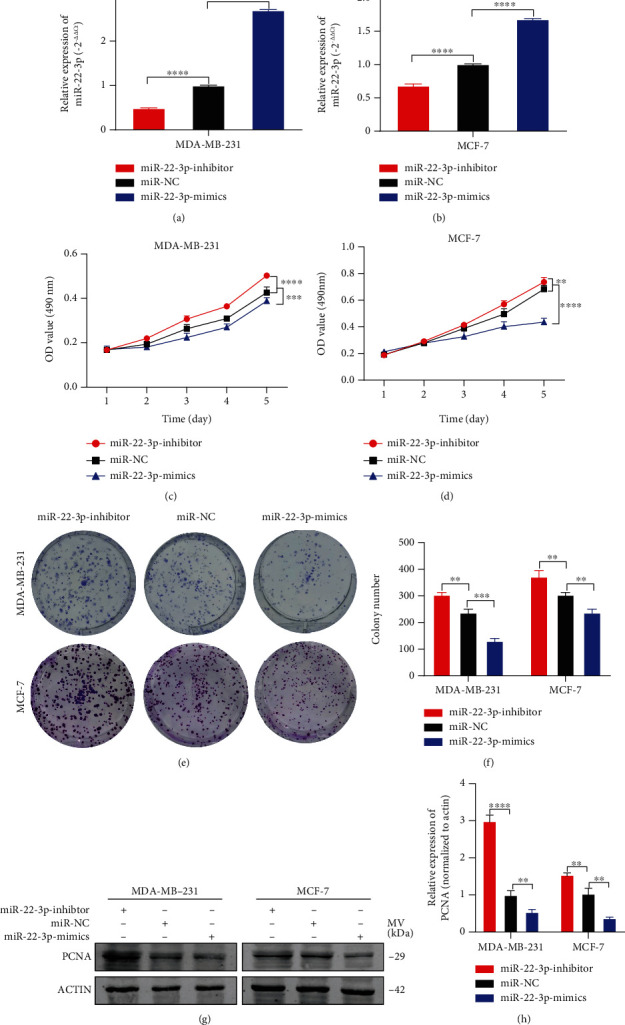
miR-22-3p suppressed cell proliferation of BC cells. (a, b) Expression of miR-22-3p was confirmed by RT-qPCR in MDA-MB-231 and MCF-7 cells. (c, d) Effect of miR-22-3p on proliferation in MDA-MB-231 and MCF-7 cells by MTT assay. (e, f) Effect of miR-22-3p on proliferation in MDA-MB-231 and MCF-7 cells by colony formation assay. (g, h) Effect of miR-22-3p on proliferation in MDA-MB-231 and MCF-7 cells by western blotting. ^∗∗^*p* < 0.01; ^∗∗∗^*p* < 0.001; ^∗∗∗∗^*p* < 0.0001.

MDA-MB-231 and MCF-7 cells were transfected with miR-22-3p mimics or inhibitor. RT-qPCR was used to verify the transfection efficiency (Figures 2(a) and 2(b)). The proliferation ability of BC cells transfected was measured by MTT assays and colony formation assays. Overexpression of miR-22-3p could suppress the proliferation of MDA-MB-231 and MCF-7 cells while miR-22-3p depletion showed opposite ability (Figures 2(c)–2(f)). Consistent with the results above, western blotting analysis demonstrated that expression of proliferation marker PCNA was inhibited by miR-22-3p mimics, (Figures 2(g) and 2(h)). All results above suggested miR-22-3p could suppress proliferation in BC cells.

### 3.3. miR-22-3p Suppressed Cell Migration of BC Cells

**Figure 3:**
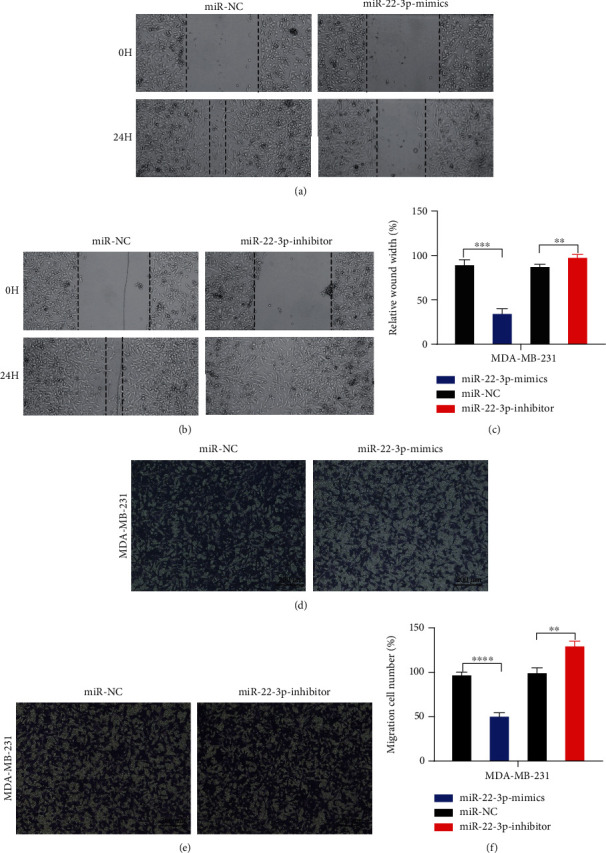
miR-22-3p suppressed cell migration of BC cells. (a–c) Wound healing assays were performed in MDA-MB-231 cell line treated with miR-22-3p mimics or miR-22-3p inhibitor (miR-NC as negative control). (d–f) Cell migration assays were performed in MDA-MB-231 cell line treated with miR-22-3p mimics or miR-22-3p inhibitor (miR-NC as negative control). ^∗∗^*p* < 0.01; ^∗∗∗^*p* < 0.001; ^∗∗∗∗^*p* < 0.0001.

We further explore the biological functions of miR-22-3p in BC migration. Through wound healing assay, limited migration was seen in the miR-22-3p high-expression group compared to the controls undergoing wound healing after 48 hours. Opposite results were observed in the miR-22-3p depletion group (Figures 3(a)–3(c)). Consistently, results of transwell migration assays showed that elevated miR-22-3p decreases cell migration in MDA-MB-231 (Figures 3(d)–3(f)).

### 3.4. PGC1*β* Is a Direct Target of miR-22-3p

**Figure 4:**
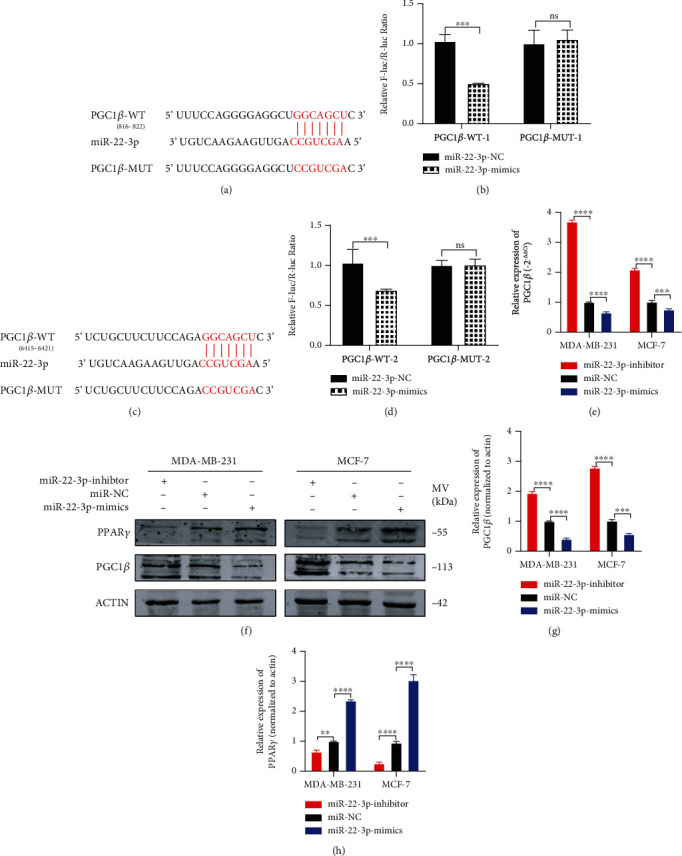
PGC1*β* is a direct target of miR-22-3p. (a, c) Putative complementary sites within miR-22-3p and PGC1*β* predicted by bioinformatics analysis (TargetScan). (b, d) Dual-luciferase reporter assays demonstrated that PGC1*β* is a direct target of miR-22-3p. (e) PGC1*β* mRNA level was determined by RT-PCR in MDA-MB-231 and MCF-7 cells with different treatment. (f–h) Representative western blots and quantification of PGC1*β* and PPAR*γ* in MDA-MB-231 and MCF-7 cells with different treatment. *β*-Actin was used as an internal control. ^∗∗^*p* < 0.01; ^∗∗∗^*p* < 0.001; ^∗∗∗∗^*p* < 0.0001.

In accordance with the prediction of TargetScan, PGC1*β* was found to be the potential target of miR-22-3p (Figures 4(a) and 4(c)). There are two possible binding sites between miR-22-3p and PGC1*β*. By constructing plasmid and mutant vectors containing 3′-UTRs with wild-type and mutant sequences, dual-fluorescein reporter assay confirmed that PGC1*β* was the direct target of miR-22-3p (Figures 4(b) and 4(d)). To verify the interaction between miR-22-3p and PGC1*β*, we detect the expression of PGC1*β* in MDA-MB-231 and MCF-7 cells transfected with miR-22-3p mimics or miR-22-3p inhibitor. The results indicated that the mRNA level of PGC1*β* was negatively regulated by miR-22-3p (Figure 4(e)). Consistently, western blotting results indicated that the protein level of PGC1*β* was significantly downregulated after transfection of miR-22-3p mimics and upregulated after transfection of miR-22-3p inhibitor (Figures 4(f)–4(h)). These results indicated that PGC1*β* is a direct target of miR-22-3p. Interestingly, when the protein level of PGC1*β* changed, PPAR*γ* showed the opposite trend. The above results prompted us to explore whether miR-22-3p/PGC1*β* suppresses BC cell tumorigenesis via PPAR*γ*.

### 3.5. miR-22-3p Suppressed the Proliferation and Migration of BC Cells via PGC1*β*

**Figure 5:**
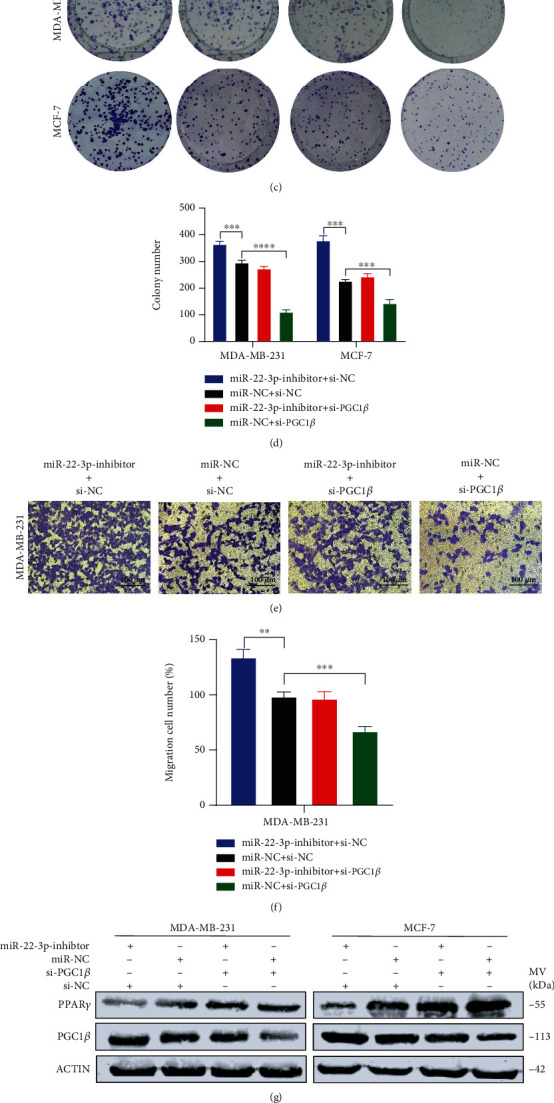
miR-22-3p suppressed the proliferation and migration of BC cells via PGC1*β*. (a–d) Knockdown of PGC1*β* partially reversed miR-22-3p inhibitor-induced promotion of proliferation in MDA-MB-231 and MCF-7 cells determined by MTT assay and colony assay. (e, f) Knockdown of PGC1*β* partially reversed miR-22-3p inhibitor-induced promotion of migration in MDA-MB-231 and MCF-7 cells determined by transwell assay. (g–i) Western blotting analysis for PGC1*β*/PPAR*γ* protein level in MDA-MB-231 and MCF-7 cells. ^∗^*p* < 0.05; ^∗∗^*p* < 0.01; ^∗∗∗^*p* < 0.001; ^∗∗∗∗^*p* < 0.0001.

We designed rescue assays in MDA-MB-231 and MCF-7 cells to further verify whether miR-22-3p affects the biological function of BC cells through PGC1*β*. After being transfected with specific siRNA of PGC1*β* (si-PGC1*β*), cell proliferation and migration ability of MDA-MB-231 and MCF-7 cells was suppressed. Meanwhile, si-PGC1*β* partially reversed the prohibitive effect of miR-22-3p inhibitor on cell proliferation and migration (Figures 5(a)–5(f)). Furthermore, the upregulation effect of the miR-22-3p inhibitor on the PGC1*β* protein level was partially inverted by si-PGC1*β* (Figures 5(g)–5(i)). Thus, we confirmed that miR-22-3p suppresses cell proliferation and migration of BC cells via directly targeting PGC1*β*.

### 3.6. Inhibition of PPAR*γ* Attenuates Suppression of miR-22-3p on BC Cells

**Figure 6:**
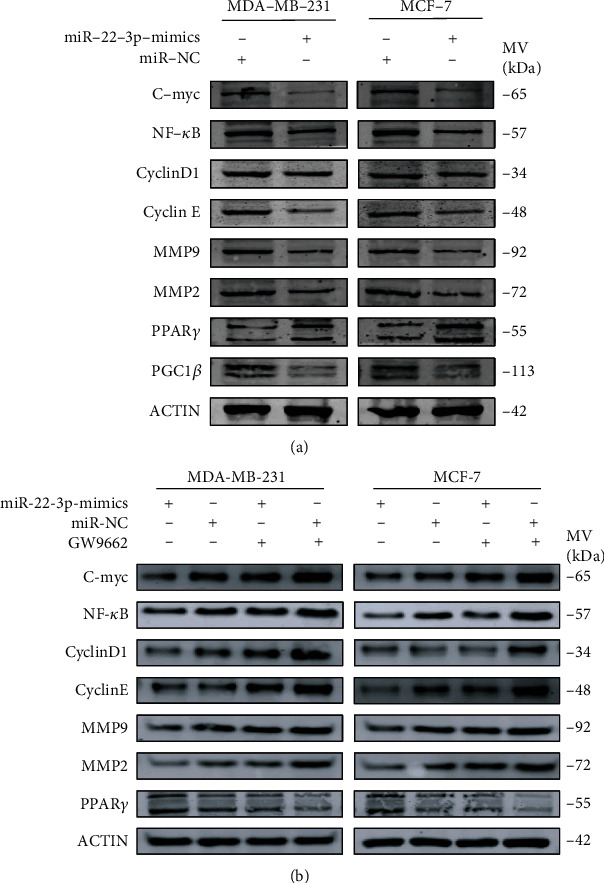
Inhibition of PPAR*γ* attenuates suppression of miR-22-3p on BC cells. (a) Upregulated miR-22-3p increased the expression of PPAR*γ* and decreased the expression of PGC1*β*, C-myc, NF-*κ*B, CyclinD1, cyclin E, MMP2, and MMP9. (b) Downregulation of C-myc, NF-*κ*B, CyclinD1, cyclin E, MMP2, and MMP9 induced by miR-22-3p was inverted by PPAR*γ* inhibition (GW9662).

Given the fact that PPAR*γ* has been reported to act as a tumor suppressor in several cancers and PPAR*γ* silencing increased the expression of C-myc, NF-*κ*B, CyclinD1, cyclin E, MMP2, and MMP9 in BC cells [28, 29]. We further explored the changes of the above factors after being transfected with miR-22-3p mimics. As expected, the protein level of PGC1*β*, C-myc, NF-*κ*B, CyclinD1, cyclin E, MMP2, and MMP9 decreased while the protein level of PPAR*γ* increased with miR-22-3p silencing (Figure 6(a)). To further prove the necessity of the PPAR*γ* signaling pathway in miR-22-3p-mediated regulations, we followed the changes of miR-22-3p overexpressing BC cells in the presence or absence of a potent specific PPAR*γ* inhibitor (GW9662). Western blot analysis showed that the downregulation of C-myc, NF-*κ*B, CyclinD1, cyclin E, MMP2, and MMP9 induced by miR-22-3p was inverted by PPAR*γ* inhibition with GW9662 (Figure 6(b)). Considering the results above, we think that the effects of miR-22-3p/PGC1*β* on BC were, at least in part, mediated by the PPAR*γ* signaling pathway.

### 3.7. miR-22-3p Suppressed BC Tumor Growth In Vivo

**Figure 7:**
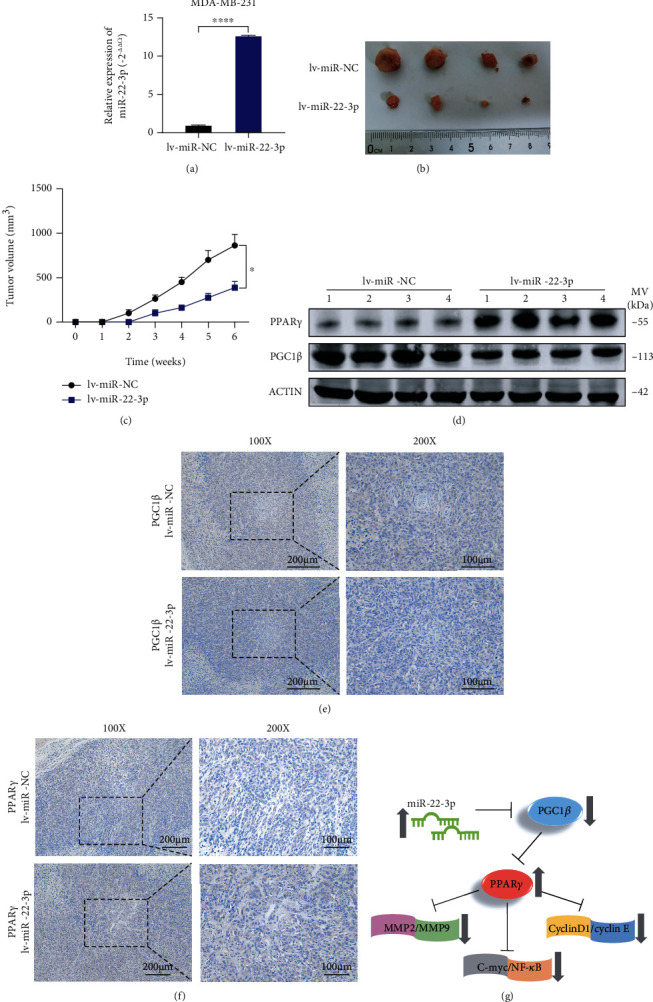
miR-22-3p suppressed BC tumor growth in vivo. (a) Overexpression of miR-22-3p in MDA-MB-231 cells was verified by RT-qPCR. (b) Representative images of xenograft tumors in nude mice. (c) The growth curves of xenografts. (d) Extract protein from tumors and measuring the expression of PGC1*β*/PPAR*γ* by western blotting. (e, f) Immunohistochemistry (IHC) staining of PGC1*β*/PPAR*γ* in xenografts. (g) The mechanism diagram was generated to illustrate the mechanism of miR-22-3p-PGC1*β*-PPAR*γ* in BC. ^∗^*p* < 0.05; ^∗∗∗∗^*p* < 0.0001.

We established a xenograft tumor model by hypodermic injection of MDA-MB-231 cells stably infected by lentivirus (lv-miR-22-3p or lv-vector) (Figure 7(a)). The tumors were collected and measured, showing that miR-22-3p could markedly decrease the tumor volume compared with the negative control (Figures 7(b) and 7(c)). Western blotting and IHC results indicated that the expression of PGC1*β* decreased while the expression of PPAR*γ* increased in the higher miR-22-3p expression group. Taking all results *in vivo* and *in vitro* together, we confirmed that miR-22-3p/PGC1*β* suppresses BC cell tumorigenesis via PPAR*γ*. The mechanism is generated in Figure 7(g).

## 4. Discussion

miRNAs have been demonstrated to be involved in various physiological and pathological processes. Here, we firstly find that the expression of miR-22-3p was lower in BC tissues than in adjacent normal tissues in TCGA dataset. Then, we found that miR-22-3p was significantly downregulated in human 47 BC samples and associated with tumor size, TNM stage, and lymph node metastasis. Overexpression of miR-22-3p markedly suppressed cell proliferation and migration of MDA-MB-231 and MCF-7 cells, indicating that miR-22-3p functions as a tumor suppressor BC. To further investigate the biological roles of miR-22-3p in BC, we demonstrated that miR-22-3p directly targets PGC1*β* by the results of the dual-luciferase reporter assays.

PGC1*β*, which has been reported to exert an important role in cancer metabolism and progression, is encoded by the gene PPARGC1*β*. Previous experimental results have confirmed that PGC1*β* was significantly overexpressed in BC. Moreover, PGC1*β* could promote proliferation and migration while inhibiting the apoptosis of BC cells, suggesting it to have a tumor-promoter role in BC [20–23]. Several studies have shown that PPAR*γ* is involved in inflammation, lipid metabolism, glucose homeostasis, and tumorigenesis [30, 31]. Specifically, recent studies showed that PPAR*γ* could inhibit cell proliferation and induces apoptosis of BC in vitro and in vivo [32–34].

To our best knowledge, this is the first study to demonstrate that the miR-22-3p/PGC1*β*/PPAR*γ* axis regulates the proliferation and migration of BC cells. Our findings suggested that PGC1*β* was directly regulated by miR-22-3p. More interesting, the protein level of PPAR*γ* increased while the protein level of C-myc, NF-*κ*B, CyclinD1, cyclin E, MMP2, and MMP9 decreased after being transfected with miR-22-3p mimics. To further prove the necessity of the PPAR*γ* signaling pathway in miR-22-3p-mediated regulations, we used a potent specific PPAR*γ* inhibitor (GW9662) in rescue assays. As expected, downregulation of C-myc, NF-*κ*B, CyclinD1, cyclin E, MMP2, and MMP9 induced by miR-22-3p was inverted by PPAR*γ* inhibition with GW9662.

Taken together, our findings suggested that the effects of miR-22-3p/PGC1*β* on BC were, at least in part, mediated by the PPAR*γ* signaling pathway. These results provided a potential novel biomarker and a therapeutic target for BC.

## Figures and Tables

**Table 1:** The relationship between the expression of miR-22-3p and various clinicopathological variables.

Patients characteristics	Total	miR-22-3p expression		*p* value^∗^
		High (*N* = 12)	Low (*N* = 35)	
Age				0.7065
<60	20	5	15	
≥60	27	7	20	
TNM stage				0.0200^∗^
I and II	30	11	19	
III and IV	17	1	16	
Tumor size (cm)				0.0237^∗^
≤2	26	10	16	
>2	21	2	19	
Lymph node metastasis				0.0423^∗^
Negative	32	11	21	
Positive	15	1	14	
Distant metastasis				0.0931
No	40	12	28	
Yes	7	0	7	

*p* value from a chi-square test (^∗^*p* < 0.05).

## Data Availability

The datasets used and analyzed during the current study are available from the corresponding author on reasonable request.

## Abbreviations

BC: Breast cancer
ncRNA: Noncoding RNA
miRNA: MicroRNA
PGC1*β*: Peroxisome proliferator-activated receptor *γ* coactivator 1*β*
PPAR*γ*: Peroxisome proliferators-activated receptor *γ*
RT-qPCR: Quantitative real-time polymerase chain reaction.

## Supplementary Materials

Supplementary Materials Figure S1: expression of miR-22-3p in TCGA database.
