# Supplementary figures and images for: miR-22-3p/PGC1β Suppresses Breast Cancer Cell Tumorigenesis via PPARγ
Source: PPAR Res. 2021 Mar 12;2021:6661828. doi: 10.1155/2021/6661828 (PMC7981180; doi:10.1155/2021/6661828)

( Fig. S1A )

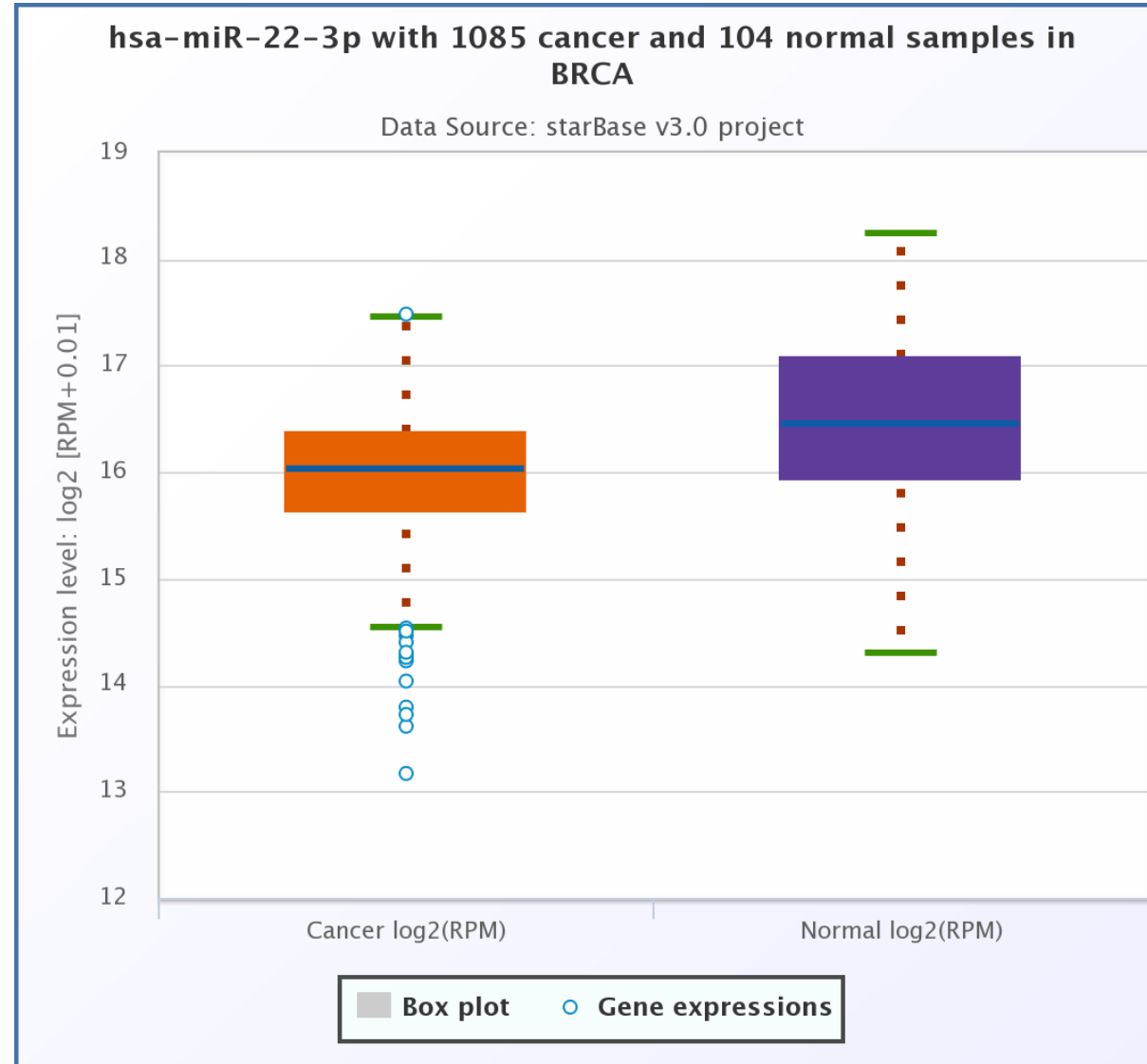

Supplement: Supplementary Materials — Figure S1: expression of miR-22-3p in TCGA database. [file 6661828.f1.pdf]
